# Genome-wide prediction of *cis*-acting RNA elements regulating tissue-specific pre-mRNA alternative splicing

**DOI:** 10.1186/1471-2164-10-S1-S4

**Published:** 2009-07-07

**Authors:** Xin Wang, Kejun Wang, Milan Radovich, Yue Wang, Guohua Wang, Weixing Feng, Jeremy R Sanford, Yunlong Liu

**Affiliations:** 1 College of Automation, Harbin Engineering University, Harbin, Heilongjiang 150001, PR China; 2 Division of Biostatistics Department of Medicine, Indiana University School of Medicine, Indianapolis, IN 46202, USA; 3 Center for Computational Biology and Bioinformatics, Indiana University School of Medicine, Indianapolis, IN 46202, USA; 4 Division of Hematology/Oncology Department of Medicine, Indiana University School of Medicine, Indianapolis, IN 46202, USA; 5 Department of Surgery, Indiana University School of Medicine, Indianapolis, IN 46202, USA; 6 Center for Medical Genomics, Indiana University School of Medicine, Indianapolis, IN 46202, USA; 7 School of Computer Science and Technology, Harbin Institute of Technology, Harbin, Heilongjiang 150001, PR China; 8 Department of Molecular, Cellular and Developmental Biology, University of California Santa Cruz, Santa Cruz, California 95064, USA

## Abstract

**Background:**

Human genes undergo various patterns of pre-mRNA splicing across different tissues. Such variation is primarily regulated by *trans*-acting factors that bind on exonic and intronic *cis*-acting RNA elements (CAEs). Here we report a computational method to mechanistically identify *cis*-acting RNA elements that contribute to the tissue-specific alternative splicing pattern. This method is an extension of our previous model, *SplicingModeler*, which predicts the significant CAEs that contribute to the splicing differences between two tissues. In this study, we introduce tissue-specific functional levels estimation step, which allows evaluating regulatory functions of predicted CAEs that are involved in more than two tissues.

**Results:**

Using a publicly available Affymetrix Genechip^® ^Human Exon Array dataset, our method identifies 652 *cis*-acting RNA elements (CAEs) across 11 human tissues. About one third of predicted CAEs can be mapped to the known RBP (RNA binding protein) binding sites or match with other predicted exonic splicing regulator databases. Interestingly, the vast majority of predicted CAEs are in intronic regulatory regions. A noticeable exception is that many exonic elements are found to regulate the alternative splicing between cerebellum and testes. Most identified elements are found to contribute to the alternative splicing between two tissues, while some are important in multiple tissues. This suggests that genome-wide alternative splicing patterns are regulated by a combination of tissue-specific *cis*-acting elements and "general elements" whose functional activities are important but differ across multiple tissues.

**Conclusion:**

In this study, we present a model-based computational approach to identify potential *cis*-acting RNA elements by considering the exon splicing variation as the combinatorial effects of multiple *cis*-acting regulators. This methodology provides a novel evaluation on the functional levels of *cis*-acting RNA elements by estimating their tissue-specific functions on various tissues.

## Background

Alternative splicing of pre-mRNA is a major mechanism of diversifying the protein coding potential of eukaryotic genomes. According to studies using large-scale expressed sequence tags (EST), as high as 60% of human genes are estimated to undergo alternative splicing [1]. Moreover, recent studies demonstrate that alternative splicing plays a pivotal role in regulating tissue-specific patterns of gene expression [2-7]. One mechanism for establishing tissue-specific alternative splicing is by modulation of the expression levels and/or intrinsic functions of "general" RNA binding proteins (RBPs) [8-10]. For instance, previous studies have reported that modulation in the relative concentrations of hnRNP A/B proteins and SR proteins can control both the alternative splice site choice and the inclusion/exclusion ratio of selected alternative exons [11-15]. In addition, another mechanism of tissue-specific alternative splicing is mediated by "tissue-specific RBPs", which accounts for the restricted expression of many RNA binding proteins to distinct tissues or developmental stages. A prototypic example is the role of Nova proteins which are crucial factors regulating brain-specific alternative splicing in the formation of synapses [16,17]. More complicated regulatory mechanisms involving the combination of large numbers of *trans*-acting RBPs (RNA binding proteins that bind to the *cis*-acting RNA elements to control pre-mRNA splicing) and *cis*-acting elements (binding sites of *trans*-acting RNA binding proteins) remain unclear and further studies are warranted.

**Figure 1:**
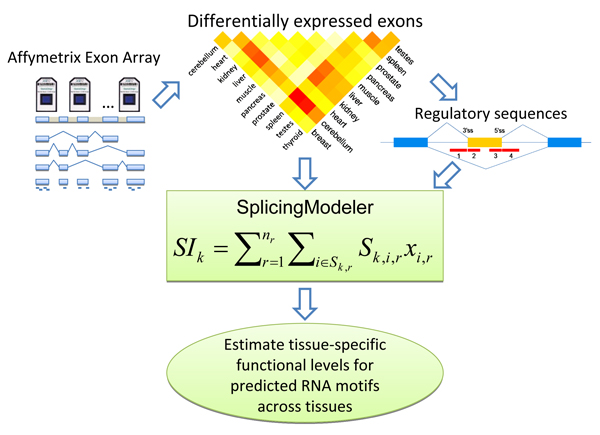
**Work flow for genome-wide *cis*-acting RNA elements prediction based on SplicingModeler and Affymetrix Human Exon Array data**.

Despite an increased focus on the factors regulating tissue-specific alternative splicing, these mechanisms still remain largely unclear. The advent of genome-wide splicing-sensitive microarrays provides a new perspective to address issues of combinatorial control of alternative splicing. Evaluation of global splicing patterns using statistical approaches has the potential to reveal how combinations of *cis*-acting RNA elements (CAEs) contribute to tissue–specific patterns of alternative splicing. These types of analytical tools are important for elucidating the combinatorial code governing splice site selection. We previously reported a model-based computational approach called *SplicingModeler*. This was successfully implemented to predict *cis*-acting RNA elements between heart and liver tissues [18]. *SplicingModeler *regards the splicing variation between two tissues as the combinatorial activities of multiple CAEs. Given the splicing index (SI) [19] of each differentially expressed exon and the number of binding sites for each motif candidate , *SplicingModeler *predicts the CAEs as well as their relative functional levels (RFL) by selecting the most significant motifs with the highest Exon Inclusion Contribution (EIC) scores. However, when being placed in a large set of tissues, we are not able to estimate CAEs' functions across tissues directly. In this study, we attempt to determine the *cis*-acting elements that contribute to tissue-specific alternative splicing and their functions across multiple tissues. Our method consists of the following steps (Figure 1): 1) For each pair of tissues, we follow a series of exon array data pre-processing procedures to identify the splicing variants; 2) The splicing index and regulatory sequences of each differentially expressed exon are then inputted into *SplicingModeler *to predict elements; 3) A two-step procedure is then performed to estimate the tissue-specific functional levels (TSFL) of predicted elements across tissues. The TSFL is a direct measure of the contribution of each CAE to inclusion or exclusion of an alternative cassette exon from an mRNA isoform in certain tissue.

We applied this method using Affymetrix GeneChip^® ^Exon Array data from 11 human tissues [20]. Without loss of generality, only cassette exon alternative splicing is considered in this study. Data from the eleven tissues resulted in 55 paired experiments which predicted 652 statistically significant *cis*-acting RNA elements (CAEs) to be involved in tissue-specific alternative splicing. Of all the predicted CAEs, the majority are located within intronic regions close to 5'splice site (ss) or 3'ss. Nearly one-third of total predicted CAEs can be mapped to known RBPs' binding sites or exonic splicing prediction databases, and another two-thirds of CAEs may be potential splicing factors. Bipartite network demonstrates that the predicted elements can be classified into two different categories; one group of CAEs functions with RBPs only in a small set of tissues, while another group plays general but differential activities across a large number of tissues.

## Results

### Differential expressions of exons among each pair of tissues

**Figure 2:**
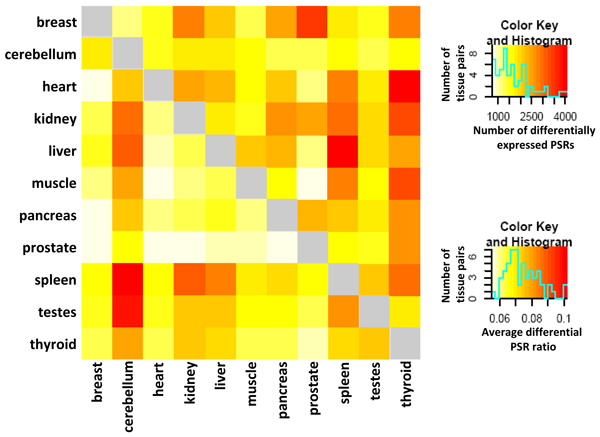
**Heatmap of differentially expressed PSRs and average differential PSR ratio per transcript for each tissue pair**. Each unit in lower left diagonal of the heatmap denotes the number of differentially expressed PSRs (exons) between row tissue and column tissue; while each unit in upper right diagonal indicates the average differential PSR ratio per transcript, calculated by the number of differential PSRs divided by total number of PSRs in each transcript. The corresponding color keys for the heatmaps and the histograms for number of differential PSRs and average differential PSR ratios are on the right side of the figure.

The Affymetrix GeneChip^® ^Exon 1.0 ST Array is designed to monitor alternative splicing using more than 1.4 million Probe Selection Regions (PSR) within 1 million exon clusters, which are constructed from various exon annotations [21]. This study is based on human exon array data incorporating 11 human tissues with 3 replicates for each available on Affymetrix website [20]. We compared the differences of PSR expressions, a fair evaluation on exon expression levels, between every pair of tissues (55 pairs in total among 11 tissues). For each tissue pair, a differential PSR ratio for each transcript is then calculated based upon the number of differentially expressed PSRs divided by the total number of PSRs in the corresponding transcript whose expression levels can be statistically detected on the array. The number of differentially expressed PSRs and average differential PSR ratios are demonstrated in Figure 2, where darker red implies greater differences in genome-wide exon expression between two tissues.

Cerebellum, spleen and testes have the largest diversities in exon expression when compared to other tissues (darker color in the lower diagonal of Figure 2). Interestingly, cerebellum and testes don't appear to have large average differential PSR ratios when compared to other tissues. Since average differential PSR ratios evaluate the average percentage of differentially expressed exons per transcript, this suggests that the alternative splicing events of these two tissues compared to other tissues is distributed broadly among transcripts. In contrast, thyroid and liver are found to have the highest differential PSR ratios but very low number of differential PSRs, which indicates that the alternative splicing events on thyroid and liver are taking place in a more specific set of genes.

### *Cis*-acting RNA elements prediction by SplicingModeler

In this study, we focus our analysis on one of the most important splicing variants, cassette exons [22], where exon inclusion and skipping leads to different types of protein isoforms. First, for each pair of tissue comparison, we use the estimation of overall gene expression to normalize the expression signals of each exon. The splicing index (SI), defined by Srinivasan K. et al [19], was then utilized to evaluate the relative quantity of splicing difference between two tissues. Second, we applied *SplicingModeler*, a computational tool we previously developed [18] to identify putative hexamers whose functional differences between two tissues potentially contribute to the differences in splicing patterns. *SplicingModeler *results in two scores for each candidate hexamer; Exon Inclusion Scores (EIC), indicating the importance of the specific hexamer; and Relative Functional Levels (RFL), where a positive or negative value suggests its role in exon inclusion in one of two comparing tissues. The most significant hexamers, which receive EIC scores that are more than 5 × IQR (interquantile range) away from median EIC score, are selected as the potential *cis*-acting elements that contribute to the splicing variations between two tissues.

**Figure 3:**
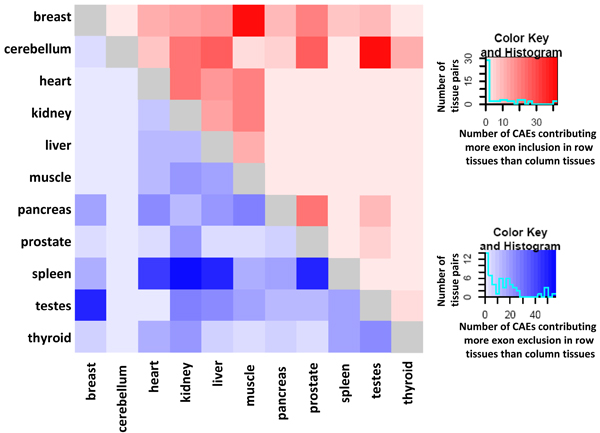
**Heatmap of predicted *cis*-acting RNA elements (CAEs) contributing more exon inclusion/exlusion to row tissues than column tissues**. For each pair of row tissue and column tissue, each unit in upper right (red colored)/lower left diagonal (blue colored) stands for the number of predicted CAEs contributing more exon inclusion/exclusion to row tissue than column tissue. Darker red and blue colors indicate larger number of predicted elements respectively.

For each pair of tissues, significant hexamers can be separated into "relative splicing enhancers" (RFL > 0) and "relative splicing silencers" (RFL < 0) depending upon their estimated functional levels. It is important to note that enhancer and silencer are relative concepts here, which describe the relative function on exon inclusion in one tissue comparing to another tissue. Figure 3 illustrates the number of identified enhancers and silencers in each pair. The upper diagonal indicates the number of identified hexamers that contribute more exon inclusion in row tissue comparing to column tissue, while the bottom diagonal demonstrates the number of hexamers that contribute more exon inclusion in column tissue comparing to the row tissue. Interestingly, most hexamers identified in cerebellum contribute to exon inclusion comparing to other tissues, while only a few are identified to be relative silencers. This trend is reversed in spleen, where most identified hexamers are predicted to decrease exon inclusion compared to other tissues. Overall, spleen, testes and pancreas are the top 3 tissues that contain more exon exclusive factors, while most predicted elements increase exon inclusion in breast and cerebellum.

### Permutation analysis

Permutation tests are applied to validate the statistical significance of predicted *cis*-acting elements. For each pair of tissues, we randomized the order of observed *SI *values, and conducted prediction using *SplicingModeler*. This approach not only effectively disconnects the functional relationships between exons and their regulatory sequences, but also preserves the regulatory element contents and the distribution of the levels of splicing variation. Wilcoxon test was then conducted on the predicted EIC scores (Exon Inclusion Score) with the alternative hypothesis that the predicted *cis*-acting elements from the original data have greater EIC scores than those from the randomized data. Permutation tests between all the tissue comparisons resulted in significant *p*-values lower than 2.2e^-16^, which suggested that *SplicingModeler *predictions are biologically meaningful.

### Biological relevance of predicted *cis*-acting elements

**Figure 4:**
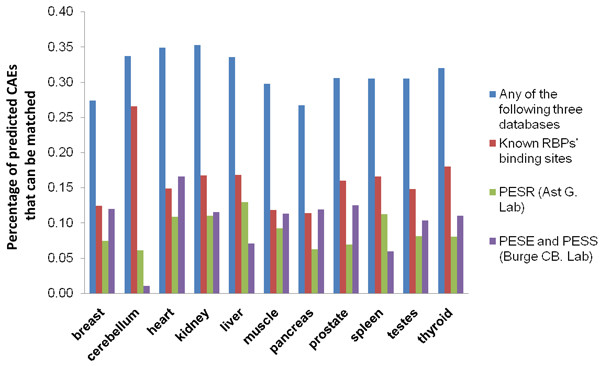
**Matching results from predicted CAEs to known RBPs' binding sites and predicted exonic splicing regulator databases**. The percentages of predicted *cis*-acting RNA elements (CAEs) matching to known RBPs' binding sites, predicted exonic splicing regulators (PESR by Ast G. Lab), predicted exonic splicing enhancers and silencers (PESE and PESS by Burge CB. Lab) and any of above three databases are represented as barplots with different colors.

In order to test the biological relevance of predicted *cis*-acting elements, we compared the sequence similarities between the predicted elements with the known binding sites of RNA binding proteins, from documented binding sites for 14 proteins [23], and predicted binding sites from splicing regulator prediction databases [24,25]. Overall, among 652 significant hexamers, 85 match with known binding sites of 7 RBPs, including hnRNP-B, hnRNP-C, hnRNP-F, hnRNP-H, hnRNP-I (PTB), SRp40 and Nova. In addition, 64 elements match with predicted exonic splicing regulators (PESR) by Ast LAB, and 60 elements match with predicted exonic splicing enhancers (PESE) and predicted exonic splicing silencers (PESS) as predicted by Burge LAB. In total, 205 of the predicted elements can be validated in any of the three databases (Figure 4).

### Functional prediction on the significant *cis*-acting elements

In order to estimate the functions of predicted elements in each tissue (not the relative functions in each tissue pair), we define Tissue-Specific Functional Level (TSFL), which is derived from the Relative Functional Levels (RFL) that are calculated for each hexamer (Equation 4) in each tissue pair. For each hexamer, the TSFL for each tissue is achieved by solving a reduced linear equation with 55 equations (total pairs of comparison) restricted by the relative relationships of 11 parameters, where each parameter models its function in the respective tissues (See Method section). The TSFL represents each hexamer's relative contribution to exon inclusion or exclusion in a specific mRNA isoform. For each element, the median value of their functions (TSFL) on exon inclusion among 11 tissues is set as baseline (0).

**Figure 5:**
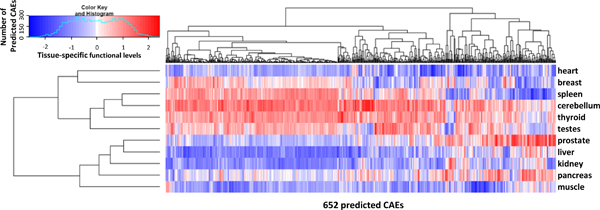
**Hierarchical clustering of tissue-specific functional levels (TSFL) for all 652 predicted *cis*-acting RNA elements (CAEs) across all eleven tissues**. TSFL greater than, lower than or equaling to 0 are represented by units with red, blue or white color. Darker red/blue indicates higher/lower functional level.

We further conducted hierarchical clustering for the 11 tissues based upon the tissue-specific functional level identified for each significant element. In principle, the splicing regulatory factors binding on these elements control global splicing pattern. Clustering tissue types based upon the functions of these regulatory elements provides unique insight in the relationship of alternative splicing across multiple tissues. The tissues were clustered using gplots package [26] of R program (, version 2.7.1) based upon Pearson's correlation between each two rows and columns (Figure 5). Two major clusters are derived. Surprisingly, high correlations are observed between liver and prostate, with a correlation coefficient of 0.415, and between spleen and cerebellum (correlation coefficient of 0.553). Further, most of the *cis*-acting elements (96.5%) are enhancing the inclusion of exons in cerebellum, and 46.6% of them contribute more exon inclusion in cerebellum than any other tissue. These data is consistent with the splicing pattern observed in Figure 2.

### Tissue-specific CAE regulatory network

**Figure 6:**
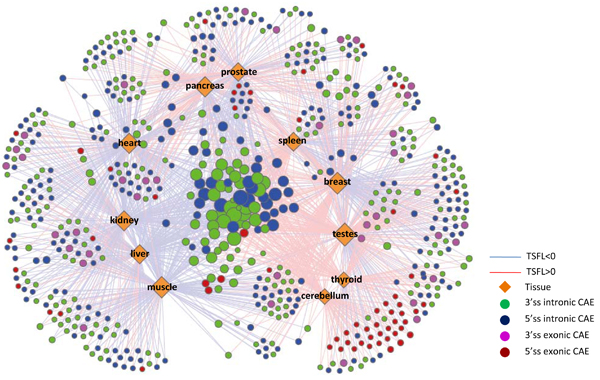
**Tissue-specific splicing regulatory network**. Yellow diamonds stand for tissues. The size of diamond is proportional to the degree of connections with *cis*-acting RNA elements (CAEs). Nodes with different colors indicate CAEs in different regulatory regions (see legends). Connections between CAEs and tissues suggest the regulatory relationships. Red and blue lines indicate TSFL > 0 and TSFL < 0, respectively. The size of circle is proportional to the number of tissues connected to CAE.

To elucidate the complex relationships between predicted *cis*-acting elements in regulating the splicing patterns across multiple tissues, a bipartite network is constructed where predicted elements and regulated tissues are considered two types of nodes (Figure 6). Each edge connecting between two types of nodes indicates the regulatory relationship between the predicted elements and the specific tissue. The color demonstrates the predicted tissue-specific functional levels.

**Figure 7:**
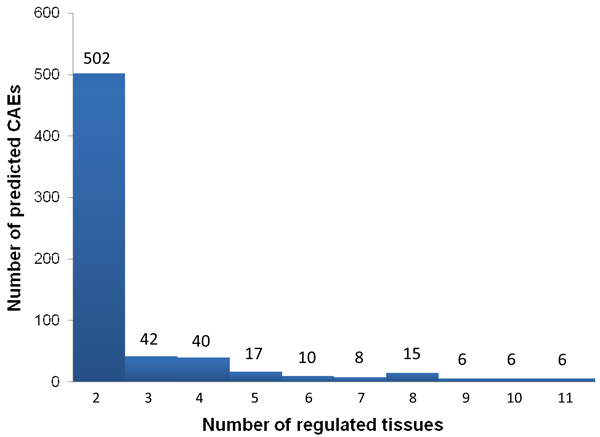
**Histogram describing the distribution of number of tissues regulated by predicted *cis*-acting RNA elements (CAEs)**.

In total, we predicted 555 intronic elements with only 97 exonic elements. Interestingly, numerous exonic factors are found in cerebellum (34/98) and testes (47/223) including PTB, hnRNP-B and hnRNP-C binding sites. In addition, a Nova binding site is found at 5'ss intronic region in cerebellum regulators. Overall, 23% of all predicted elements regulate multiple (>= 3) tissues, although most of the elements only connected to two tissues (Figure 7).

## Discussion

In this study, using an extended version of *SplicingModeler*, we successfully identified 652 *cis*-acting RNA elements in 11 human tissues that are important in regulating alternative splicing. Permutation tests for all experiments suggest that the predicted *cis*-acting elements are statistically significant and biologically relevant. Thirteen percent of predicted elements can be mapped to known binding sites of RNA binding proteins, and 31.4% can be matched in any one of the three databases that document biologically-determined known sites and predicted sites using other bioinformatics approaches.

Previous studies on splicing regulation tends to focus on exonic regions, our results suggest that intronic regions may also be equally, if not more, important for the regulation of tissue-specific alternative splicing. Consistent with previous observations [27-29], the majority of *cis*-acting elements predicted are in intronic regions, and only 23% are in exonic regions. An important exception, of all 38 elements regulating alternative splicing between cerebellum and testes, 32 are exonic regulators. Interestingly, of all the exonic regulators in cerebellum and testes, only one contributes more exon inclusion in testes than cerebellum, indicating that a large set of exonic *cis*-acting elements are regulating exon skipping in testes. Although alternative splicing is frequently observed in human brain and testes [3,6,30], the differentially expressed exons (Figure 2) show that these two tissues undergo different splicing patterns. Compared to other tissues, more exonic elements are found to regulate alternative cassette exons in testes.

Different from relative functional levels, derived from *SplicingModeler *to estimate the relative activities of *cis*-acting RNA elements for each pair of tissues, tissue-specific functional levels are developed in this study to represent the activities of predicted *cis*-acting RNA elements on exon inclusion in one tissue. From the hierarchical clustering on the tissue-specific functional levels of predicted *cis*-acting elements across all tissues, we observe positive correlations within each of the two major clusters. The correlation coefficients between cerebellum and spleen, and between liver and prostate, even reach high values of 0.553 and 0.415 respectively, indicating that a large number of *cis*-acting RNA elements are playing similar functions across these tissues.

From bipartite network showing the regulatory relationship of predicted *cis*-acting elements (CAE) in different tissues (Figure 6), we clearly see that most of the predicted CAEs (small circles surrounding tissues) are only connected to two tissues; meanwhile, a small number of CAEs (big circles centered and surrounded by tissues) are regulating multiple tissues. We also demonstrate that the vast majority of predicted CAEs are only related to two tissues (Figure 7). Interestingly, 14 CAEs are predicted to be Nova binding sites, and all of them are only significantly regulating the splicing differences between two tissues. Similar situations also happen on SRp40, hnRNP-F and hnRNP-H related CAEs, in which 88.9%, 75% and 100% are found to regulate only two tissues respectively. This is either caused by the degenerative features of these binding sites, which is not considered in the current model, or implies that the binding sites of these general splicing factors slightly vary in different tissues. Interestingly, 44.6% of all hnRNP-B, hnRNP-C and hnRNP-I (PTB) related CAEs are significantly presented in multiple (>= 3) tissues. These data indicate that the majority of CAEs may be only recognized by tissue-specific RBPs and only function in a small set of tissues. By contrast, a small number of CAEs bound by highly abundant RBPs like hnRNP-B/C/I, could also contribute to the differential regulation of alternative splicing across multiple tissues.

Systematic investigation of RNA binding proteins and their complicated interactions and regulatory roles on alternative splicing remains a challenging problem. Computational models integrating genome-wide expression data can assist in the identification of mechanisms influencing tissue-specific alternative splicing. Popular computational methods of *cis*-acting RNA elements prediction are typically based on low- or high-throughput microarray or sequencing data, and then analyzed to predict binding sites using the consensus sequences of the studied RBPs [24,31-33]. Such methods are useful for predicting binding specificity of specific RBP's, but lack integration of tissue-specific splicing and combinatorial regulatory mechanisms. Moreover, these methods are mainly restricted to exonic splicing regulator prediction. Recently, Das et al developed another computational model to identify CAEs for tissue-specific alternative splicing, which uses the correlation of motif parameters together with gene-level normalized exon expression signals to identify splicing regulatory motifs [34]. However, combinatorial effects of different splicing factors were not considered. Besides, only upstream and downstream intronic regions are analyzed in that study, however, exonic splicing enhancers (ESE) and silencers (ESS) are also important parts of alternative splicing regulators as well. Our method is based on the assumption that alternative splicing is regulated by RBPs in a combinatorial fashion, and therefore potentiates an unbiased investigation on the functions of *cis*-acting elements in both intronic and exonic regions.

Compared with previous computational methods for the prediction of CAEs regulating tissue-specific alternative splicing [24,31-34], our method attempts to predict *cis*-regulatory elements in combination with their functions and contributions to exon inclusions. Different from *SplicingModeler*, this study emphasizes the tissue-specific rather than relative functional levels between tissues, so that we are able to compare CAEs' activities in a broader scale. Further improvements of our model in future include sequence degeneracy of motif candidates and pre-mRNA secondary structure.

## Methods

### Data source

Human Exon Array dataset, containing exon array data for 11 tissues (including breast, cerebellum, heart, kidney, liver, muscle, pancreas, prostate, spleen, testes, and thyroid) with 3 replicates for each, were obtained from Affymetrix Exon 1.0ST Array Sample Dataset [20]. Affymetrix Power Tool [35] (APT Release 1.8.5) were used to pre-process exon array data such as probe intensity normalization, probeset and transcript expression summarization, implementation of MiDAS algorithm [36] etc. Since our analysis focuses primarily on cassette exons, Affymetrix core PSR dataset, which contains annotated RefSeq transcripts and full-length mRNAs, were used to filter out exon clusters with multiple PSRs. UCSC known gene database and annotation files [37] were retrieved from UCSC Genome Browser.

### Identification of differentially expressed exons

For each pair of tissues ( pairs in total), we identify the differential expressed exons, following the workflow suggested by Affymetrix technique note [21]. First, probe intensities are adjusted based upon the median intensity of background probes with similar GC content. Quantile-normalization is then applied within tissues on the assumption that probe intensities in different replicates of same tissue follow the same distribution. Intensities of probesets and transcripts are then summarized based on Plier [38] and Iter-Plier [39] methods respectively. For each sample, PSRs with DABG (Detection Above Background) p-values [40] less than 0.05 are treated as present. For each tissue type, PSR is regarded as present across replicates if it is present in more than two replicates of a tissue. PSRs that are absent in one of the pair of tissues will be filtered out. Transcript is regarded as present if more than 50% of all PSRs are present. Transcripts not expressed in both tissues are removed; this effectively removes the false positive identification of alternatively spliced exons due to unbalanced gene expression. MiDAS algorithm [36] is then used to test the significance for each present PSR to be differentially expressed; PSRs with p-values less than 0.05 are considered as differentially expressed. In order to compare the exon inclusion rate in two tissues, Splicing Index (SI) is calculated based on the gene-level normalized intensities (NI) [19].

(1)

Exons with |SI|>= 2.0 are selected as splice variants between these two tissues; this can be translate into four times splicing difference.

### Regulatory sequences

It has been reported that splicing factors works primarily in exonic and intronic regions that are adjacent to splice sites [41-43]. As such, *SplicingModeler *only considers intronic regions within 300-bp and exonic regions within 150-bp to 5' and 3' splice sites as four potential *cis*-regulatory regions. *SplicingModeler *requires the SI values as well as regulatory sequences to perform the prediction of regulatory *cis*-acting elements. The differentially expressed exons are mapped to UCSC known gene annotation [37] to obtain their genomic loci. Sequences in regulatory regions, including intronic/exonic regions upstream and downstream of 5' and 3' splice sites, are then retrieved from Human Genome Database (hg18) [44,45].

### *Cis*-acting RNA elements prediction based on SplicingModeler

*SplicingModeler *[18] is conducted to predict critical *cis*-acting elements that contribute to splicing pattern differences between each of  tissue pairs. It is designed to predict *cis*-acting RNA elements based on the regulatory sequences and corresponding SI values of differentially expressed exons. The model regards the exon inclusion variations between two tissues as the combinatorial effects of multiple splicing factors. The quantitative relationship between SI value of the *k-th *exon and occurrences of candidate hexamers is modeled as:

(2)

where, *n*_*r *_indicates the number of regulatory regions (*n*_*r *_= 1 to 4 corresponding to 3'ss-intronic region, 3'ss-exonic region, 5'ss-exonic region, and 3'ss-intronic regions); *S*_*k*, *r *_is the total set of functional candidate elements in regulatory region *r*; *S*_*k*, *i*, *r *_is the number of binding sites of candidate element *i *in regulatory *r-th *region on the *k-th *exon; *RFL*_*i*, *r *_stands for the relative functional level of motif *i *in regulatory region *r*; a positive and negative value implies its function on exon inclusion in one of the two comparing tissues.

*S*_*k*, *i*, *r *_can be obtained from the regulatory sequences, *SI*_*k *_stands for the splicing index of the *k-th *exon, which will be measured by the exon array experiment, and calculated based upon Equation 1. The Relative Functional Level (RFL) can then be estimated by fitting *k *equations with *n*_*r *_parameters using least-squares procedure. The significant *cis*-acting elements are selected in an iterative fashion. In brief, *n *= 20 candidate elements were randomly selected from all the candidates. The adjusted reciprocal of sum square error, calculated following least-squares procedure, is equally deposited to exon inclusion contribution (EIC) score of each motif candidate . The estimated RFL values are also accumulated into the final RFL scores. A detailed description on the *SplicingModeler *procedure can be found in [18].

In this study, to speed up the modeling, motif candidates whose binding sites occurrences are less than 5% of all alternatively spliced exons are filtered out. For each tissue pair comparison, we repeat the procedure for 10 million times. Motif candidates whose EIC scores are higher than median + 5 × IQR (interquantile range) are selected as significant *cis*-acting elements.

### Tissue-specific functional levels estimation for predicted *cis*-acting element

Functional levels of predicted elements in each tissue, or Tissue-Specific Functional Level (TSFL), is derived from the Relative Functional Levels (RFL) that are calculated for each hexamer (Equation 4) in tissue pairs. The TSFL is calculated through the following two steps:

#### Normalize relative functional levels across 11 tissues

For each tissue pair comparison, the derived RFL for the *m-th *element is proportional to the instances that motif candidate *m *is selected in the *SplicingModeler *procedure. This number could be different since the hexamers whose binding sites occurrences are less than 5% of all alternatively spliced exons are pre-filtered out. The normalization procedure is conducted to eliminate this bias. If there are totally *M*_*i*, *j *_candidate motifs were used in the *SplicingModeler *when comparing paired tissues *i *and *j*, the normalized relative functional level for motif candidate *m *can be calculated using following formula:

(3)

#### Estimate tissue-specific functional levels

If *TSFL*_*m*, *i *_represents the functional level of motif *m *for tissue *i*, the relative functional level (RFL) of the same motif between tissues *i *and *j *can be reconstructed as:

(4)

For any motif candidate , such equation exists for every tissue comparison. Based on *nRFL*_*m*, *i*, *j *_score estimated from *SplicingModeler*, *TSFL *value was solved using least-squares procedure by randomly selecting one tissue as the standard and assigning 0 to its corresponding TSFL. For every predicted motif, the derived TSFL score is further normalized assuming standard normal distribution.

### Bipartite splicing regulatory network

To illustrate regulatory relationships of predicted *cis*-acting RNA elements (CAEs) on regulated tissues, a bipartite network is constructed using Cytoscape [46]. Predicted CAEs and tissues are represented by circular and diamond-shaped nodes respectively. Different colors of circles stand for different types of regulatory regions (green: intronic region adjacent to 3'ss; blue: intronic region adjacent to 5'ss; aubergine: exonic region adjacent to 3'ss; red: exonic region adjacent to 5'ss). The size of CAE is proportional to the number of connected tissues. CAEs connected to multiple tissues are clustered at the center of graph, while CAEs only associated with two tissues are located at surrounding regions. The connection between each CAE and tissue indicates that there is a regulatory relationship between them. Colors of connections for each CAE describe the tissue-specific functional levels (TSFL) across tissues (Red: TSFL > 0; Blue: TSFL < 0).

## Competing interests

The authors declare that they have no competing interests.

## Authors' contributions

XW and YL contributed to the design of the study. XW and YL designed and performed the computational modelling and drafted the manuscript. KW, MR, JS, YW, WF and GW participated in coordination, discussions related to result interpretation and revision of the manuscript. All the authors read and approved the final manuscript.
